# Nanomaterials in Sensors

**DOI:** 10.3390/nano3040572

**Published:** 2013-10-14

**Authors:** Joseph J. BelBruno

**Affiliations:** Department of Chemistry and Center for Nanomaterials Research, Dartmouth College, Hanover, NH 03755, USA; E-Mail: jjbchem@dartmouth.edu; Tel. +1-603-646-2501; Fax: +1-603-646-3946

This Special Issue of *Nanomaterials* is focused on the continuing implementation of nanomaterials and nanostructures in the development of more sensitive and more specific sensing devices. As a result, these new devices employ smaller sensing elements and provide more “real time” capability. Often, the inclusion of nanomaterials leads to sensing elements for targets that were previously inaccessible. The nanostructures employed in sensor development include (among others): nanowires, semiconductor particles, various allotropes of carbon and imprinted polymeric spheres. Nanoparticles, in general, exhibit physical properties that not only differ from the parent bulk material, but also from other nanoparticles that are of different dimensions. This uniqueness offers more opportunity to fine-tune a sensor in order to discriminately detect one component of a complex mixture. The reports in this issue of the journal utilize the properties of nanomaterials to create novel sensing systems.

Huang and Liu [1] describe the use of graphene oxide to selectively adsorb single-stranded DNA from a mixture of single- and double-stranded material. While not a sensor, *per se*, the utility of this procedure to those wishing to monitor one type of biomaterial in a mixture of DNA, is clear and was shown to result in a purified solution of double-stranded DNA. We should expect reports utilizing the technique in sensing to appear in the near future.

Hijiri *et al*. [2] report on the use of ZnO nanostructures to selectively monitor CO and NO_2_ in air. This interesting study compared the relative response of prismatic nanoparticles annealed at two very different temperatures to the two atmospheric pollutants. High temperature annealing led to a selective CO sensing element, while a lower temperature treatment provided material that could not differentiate between the two analytes. Electrospun fibers, on the other hand, were found to be ideal for the selective detection of NO_2_.

Laminack and Gole [3] have studied the functionalization of metal oxides supported on porous silicon to provide information regarding a system that may be tuned to a particular target molecule. Nanostructured TiO_2_, SnO*_x_*, NiO and Cu*_x_*O were deposited and treated with nitrogen or sulfur donating molecules. By means of their new inverse hard and soft acids and bases (IHSAB) model, the authors provide a guide to the development of new sensing materials.

In a review, Song and Choi [4] describe the specific use of conductive polyaniline (PANi) nanowires as the active layer for chemiresistive sensors. This comprehensive manuscript includes reports on the synthesis, structure and reactions of PANi nanowires. The myriad ways of incorporating the polymeric nanowires into sensing systems are reviewed, and the current limitations (which provide a rich source for future research), are noted.

A more general review, contributed by Yoon [5], looks at trends in a range of conducting polymer nanomaterials employed as sensing elements. The reviewed materials include polypyrrole, polyaniline, polythiophene and poly(3,4-ethylenedioxythiophene). The authors provide a detailed listing of production methods leading to different morphologies, as well as a range of chemical and biological sensor applications.

The range of sensor applications and the wide variability in sensor properties discussed in this issue indicates that the application of nanomaterials in sensing technology, a relatively new field of research, has a healthy and promising future. I encourage you to read through this Special Issue and use the valuable information provided therein to help us move forward in this exciting area.
